# Driving Skills of Individuals With and Without Developmental Coordination Disorder (DCD/Dyspraxia)

**DOI:** 10.3389/fnhum.2021.635649

**Published:** 2021-03-08

**Authors:** Judith Gentle, Daniel Brady, Nigel Woodger, Sophie Croston, Hayley C. Leonard

**Affiliations:** ^1^School of Psychology, University of Surrey, Guildford, United Kingdom; ^2^School of Psychology and Clinical Language Sciences, University of Reading, Reading, United Kingdom

**Keywords:** developmental coordination disorder/dyspraxia, driving, motor coordination, perception, experience, real-world skills

## Abstract

Learning to drive is a significant event for the transition to adulthood and delay or avoidance may have social, practical, and psychological implications. For those with Developmental Coordination Disorder (DCD/Dyspraxia), driving presents a considerable challenge, and the literature shows that there are differences in driving ability between individuals with and without DCD. The aim of the current research is to further our understanding of the mechanisms underlying the driving experiences of individuals with DCD. Nineteen participants with DCD (10 drivers and 9 non-drivers) and 36 controls (17 drivers and 19 non-drivers) aged 18–57 years took part in this study. Participants completed standardized tests, questionnaires and a driving simulation task designed to measure speed, road positioning, and rate of change of steering in three conditions with increasing perceptual complexity. Results indicate that behaviors for all participants changed as the perceptual demands of the task increased. However, drivers with DCD were more affected than all other groups, driving more slowly, and driving further to the right. These findings illustrate how the impact of both internal and external constraints negatively affect the success of the driving task for individuals with DCD compared to their TD peers.

## Introduction

Driving is a core skill necessary for access to many activities of daily living. For example, drivers have independence and flexibility, increasing access to opportunities for education, employment, and leisure compared to non-drivers (Barkley, 2004). Furthermore, learning to drive is a significant event for the transition to adulthood and delay or avoidance may have social, practical and psychological implications (Barkley, 2004; Kirby et al., 2011). However, driving is not a simple task. Drivers must have good spatial perception (de Oliveira and Wann, 2011) and well-coordinated visuomotor control (Marple-Horvat et al., 2005) to be able to manage multiple stimuli simultaneously and react quickly in an emergency when completing the driving task (Reger et al., 2004).

In order to interpret environmental constraints and elicit skilled actions (e.g., to steer a car around obstacles in the pathway), humans are heavily dependent upon accurate perception (Hulme and Snowling, 2009), particularly vision and proprioception. Additionally, cognitive input is necessary to process the feedback and feedforward information in response to the environment and to allow predictions about the expected consequence of a motor command (Wolpert et al., 2003). Research by Newell (1986) considers how constraints within the individual (flexibility, strength, motor, or sensory systems), the task (moving/stationary, accuracy/speed, and seated/standing) and the environment (environmental stability, lighting, and surface type) affect behavior. Thus, movement is viewed as an emergent process arising from dynamic interaction between the individual, the task and the environment. When all these systems integrate efficiently with motor control mechanisms, the individual can adapt to dynamic environmental constraints.

However, whilst driving the ability to accurately process sensory information from the environment may be compromised as this information has to be perceived at much higher speeds than humans are designed to travel. Additionally, cars are being designed with ever-more luxurious interiors such as improved soundproofing (to reduce external noise) and improved suspension (to increase smoothness of the ride, Davies, 2017). It is therefore possible that whilst these advances certainly make the driving experience more pleasurable, they may negatively affect the ability to use sensory information effectively and make informed judgments about the external environment. This could have a particular impact on individuals with sensorimotor difficulties, as is outlined below.

### Driving With Developmental Coordination Disorder

Developmental Coordination Disorder (DCD/Dyspraxia) is a neurodevelopmental movement disorder that affects the development of motor control and coordination but is unexplained by a neurological condition. Prevalence rates are estimated to be around 5% of children aged 5–11 years (American Psychiatric Association [APA], 2013) and the motor impairments continue to negatively impact everyday activities into adulthood (Purcell et al., 2015). Individuals with DCD are known to have difficulties with sensori-perceptual function (visual form detection, motion detection, visuospatial processing, and tactile perception), forward modeling (Wilson et al., 2013), perception-action couplings (Wann et al., 1998), and learning new motor tasks (Missiuna et al., 2008). It is therefore unsurprising that learning to drive has been identified as an area of particular difficulty for this population (Cousins and Smyth, 2003; Missiuna et al., 2008; Kirby et al., 2011; Blank et al., 2019).

There is a paucity of research investigating the effects of sensori-perceptual processing on environmental interactions for individuals with DCD. However, research investigating behaviors of pedestrians show that adults with DCD are slower than controls when negotiating obstacles in their pathway (Cousins and Smyth, 2003; Wilmut et al., 2015; Gentle et al., 2016). The additional time taken to adjust to environmental constraints could indicate slower perceptuo-motor integration, i.e., individuals with DCD need more time to perceive relevant information and adopt the appropriate motor actions to avoid collisions (Wilmut et al., 2015; Gentle et al., 2016). Indeed, a real-time study investigating road crossing skills found that children with DCD did not allow enough time to safely cross the road. The authors argue if this outcome were translated to a real-world environment, a collision would have occurred (Purcell et al., 2017). These findings reflect anecdotal evidence of collisions with obstacles for pedestrians with DCD (Geuze, 2007) and have important implications for driving, where the time to process relevant information is shorter due to the higher speed of travel. Another observation of pedestrian behaviors relevant for driving is that individuals with DCD turn more often and to a greater degree than their typical counterparts when passing through a narrow aperture (Wilmut et al., 2015). The authors argue that these adaptations accommodate motor control difficulties, and this behavior ensures a wider margin when passing through the aperture thus avoiding a collision. Exactly how this adaptation transfers to a real-world driving task where narrow roadways are a common occurrence, has yet to be investigated for individuals with DCD.

Driving research shows that fewer individuals with DCD learn to drive compared to their typically developing (TD) peers (Kirby et al., 2011), and those who do succeed take longer to pass their test (Missiuna et al., 2008). Individuals with DCD also perceive themselves to be less competent at driving and report particular difficulty with more complex skills, such as parking or reversing (Missiuna et al., 2008; Kirby et al., 2011). Research using an automatic car simulator found that individuals with DCD used significantly more steering adjustments when maintaining a straight course at a controlled steady speed, used twice as many steering adjustments as necessary when negotiating a bend and had slower responses to pedestrians in their pathway than their age-matched TD peers (de Oliveira and Wann, 2011, 2012). de Oliveira and Wann (2012) further identified that those with DCD showed a larger variance in heading when turning bends but not when driving along straight roads compared to their TD peers. The authors explain their findings in terms of deficient mapping between visual information and steering actions (Fajen, 2008). Finally, de Oliveira et al. (2014) used a steering task to investigate the use of advanced visual information. They found that, whilst TD individuals showed a linear improvement as duration of visual information increased, individuals with DCD showed behaviors described as U-shaped, where optimal performance occurred with 750 ms of advance information. However, studies from the de Oliveira group collected data from young (mean age 17.4 years, 2011; 18.6 years, 2012; and 19 years 2014) and inexperienced drivers. Furthermore, the driving simulator used by de Oliveira and Wann comprised a simulator chair and steering wheel which is perhaps more indicative of a computer game than a real car. It is unclear whether older drivers would show similar behavior in a more ecologically valid simulator.

The aims of the current study were to extend the small body of previous research by investigating competencies of participants with and without DCD when negotiating everyday driving scenarios in conditions not previously tested. The use of a real car driving simulator (compared to the use of a chair, steering wheel and pedals used by de Oliveira and Wann, 2011, 2012) enhances ecological validity and the generalizability of the findings.

Based on the previous research we aimed to design an ecologically valid set up, using frequently experienced driving settings, to address three research questions: (1) Do drivers and non-drivers (defined by driving test status) with and without DCD behave differently when processing dynamic sensory information in progressively complex environments? (2) How do drivers and non-drivers with and without DCD negotiate narrow apertures in a car of fixed width? (3) Do individuals with DCD collide more with obstacles in the pathway compared to controls?

This experiment consisted of three conditions comparing speed, road positioning and steering adjustments between the groups and in scenarios of increasing perceptual load. *Condition 1* was a low load condition as drivers negotiated a clear, straight road; *Condition 2* increased the perceptual load as participants negotiated a clear, straight road, with the addition of stationary objects in the pathway; *Condition 3* increased the perceptual demands further as participants negotiated a clear, straight road, with the addition of an oncoming moving vehicle.

Research question 1 was addressed through all three conditions. We hypothesized that across all conditions, compared to typical drivers, individuals with DCD would drive more slowly, have less appropriate road positioning and use more steering adjustments and compared to typical drivers, typical non-drivers would drive more slowly throughout. Furthermore, we predicted that these differences would be more pronounced in conditions with higher perceptual load. Research Question 2 was examined in Condition 1 as participants drove along a road with cars parked either side. We hypothesized that, compared to typical drivers, non-drivers,and individuals with DCD would drive more slowly, have less appropriate road positioning and use more steering adjustments during this condition. Finally, research Question 3 was investigated in Conditions 1 and 2. We hypothesized that, compared to typical controls, participants with DCD would have more collisions during the drive.

## Method

This research was approved by the University of Surrey Research Ethics Committee and informed consent was obtained from all participants.

### Participants

Table 1 presents demographic information for the current sample. Nineteen individuals with DCD (10 drivers and 9 non-drivers; mean age: 26.5 years) and 36 controls (17 drivers and 19 non-drivers mean age: 21 years) participated in this study. Participants with a valid driving license were assigned to the Drivers group, participants without a valid drivers license were assigned to the Non-drivers group. All participants with DCD were recruited in line with the DSM-5 (American Psychiatric Association [APA], 2013) and the United Kingdom guidelines for assessment of adults with DCD (Barnett et al., 2015). Participants with DCD were recruited through a charitable foundation which supports individuals with DCD and contacts known to the researchers. Additionally, an advertisement was placed in the university setting to recruit those individuals with a diagnosis of DCD who have not taken part in previous research with the research team as well as TD controls.

**TABLE 1:** Means of demographic data for participants in this study.

Measure	DCD group (*N* = 19)	DCD (*SD*)	TD group (*N* = 36)	TD (*SD*)	*p* value
Age	26.5y	(2.38)	21y	(0.92)	*p* = 0.017*
Gender ratio M: F (17:38)	9:10		8:28		*p* = 0.027*
Drivers: Non-drivers (27:28)	10:9		17:19		*p* > 0.005
M-ABC-2 (percentile)	6.07	(1.68)	46.9	(3.43)	*p* < 0.001*
ADC	75.2	(15.29)	17.2	(18.19)	*p* < 0.001*
CAARS	53.83	(2.96)	48.7	(2.12)	*p* > 0.005
WIAS-IV Vocab	27.6	(4.51)	39.1	(4.42)	*p* = 0.112
WIAS-IV Block design	33.7	(2.97)	42.9	(1.68)	*p* = 0.005*

*Significant group differences (TD, DCD) for each measure are also reported.*

### Materials

#### Measures to Assess DCD

A range of assessments were used to ensure that the four DSM-5 diagnostic criteria for DCD were met. To assess coordinated motor skills (Criterion A) participants completed the Movement Assessment Battery for Children (MABC-2, Henderson et al., 2007), which is a standardized measure of motor skill suitable for ages 3–16 years. Due to the lack of appropriate motor assessments in the United Kingdom for adults with DCD, the 11–16-year age band was used, reflecting common practice in DCD research with adults (Cousins and Smyth, 2003; Cantell et al., 2008; Purcell et al., 2015). Individuals scoring below the 5th percentile demonstrate severe motor difficulties, and those scoring at or below the 15th percentile demonstrate moderate motor difficulties.

The Adult DCD checklist (ADC, Kirby et al., 2010) was used to assess Criterion B (motor deficit significantly interferes with daily living activities). The ADC is a standardized screening tool for those over the age of 16 to aid identification of DCD in adults. A score of at least 17 in section 1 of the ADC and a total score of at least 56 is required to meet DSM-5 criteria and demonstrates a significant effect of motor difficulties on everyday life which has been present since childhood (Criterion C). Participants were asked whether they had any visual impairment or neurological condition that would explain any movement difficulties (Criterion D). Participants were also tested using the Wechsler Adult Intelligence Scales (WAIS-IV, Wechsler, 2010) to provide a measure of verbal IQ. Participants were assigned to the DCD group if they scored above the cut-offs identified above on the ADC, below the 15th percentile on the MABC-2, had no visual impairment or neurological condition, and a verbal IQ score in the typical range. Participants were assigned to the control group if they scored within the typical range on the ADC, MABC-2, and verbal IQ, with no visual impairment or neurological condition.

Finally, participants were asked to complete the Conners Adult ADHD Scales, Short version (CAARS-S:SV, Conners et al., 2000). The CAARS-S:SV consists of 30 statements which relate to symptoms or behaviors associated with Attention Deficit-Hyperactivity Disorder (ADHD), which often co-occurs with DCD. Participants rate themselves in relation to each of these statements on a scale of 0–3, with a higher number corresponding to a higher frequency of the particular symptom (0 = not at all, 1 = just a little, 2 = often, and 3 = very frequently). Participants scoring highly on the CAARS (T-Score > 60) were not excluded as running the statistical analysis with and without them did not affect the results.

#### The Driving Simulator

As data were collected in the United Kingdom, speed limit signs and measurements are in miles per hour. Metric equivalents are provided with United Kingdom measurements provided in brackets throughout.

**FIGURE 1:**
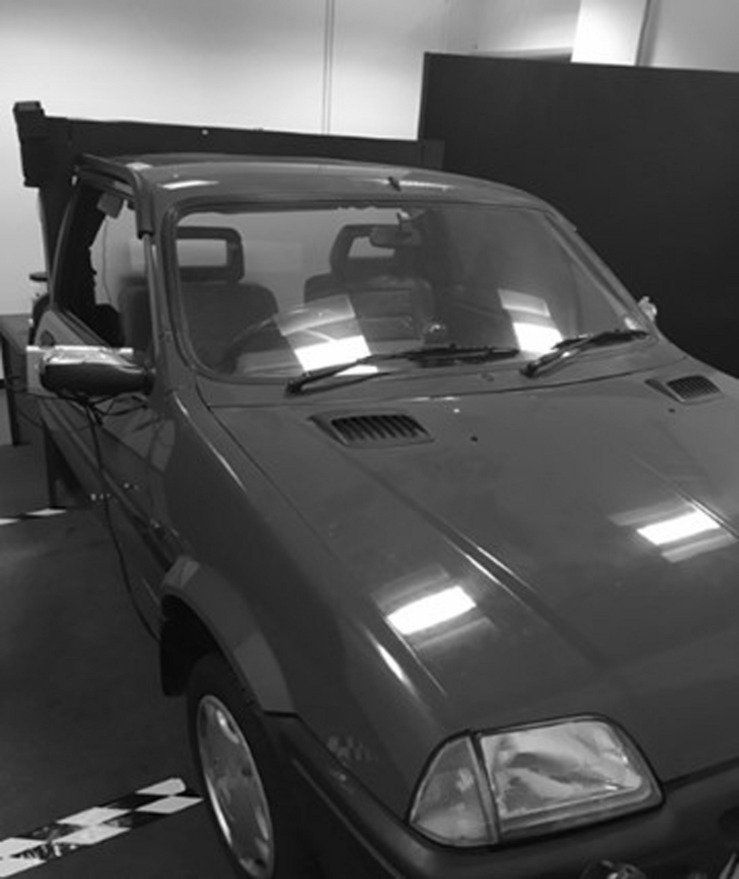
Photo of the car simulator.

The driving task was presented in a driving simulator (SIM. Systems Technology Incorporated STISIM Drive; see Figure 1). This method allows participants to drive in a real car on a virtual road and creates a naturalistic experience where drivers can turn their head to look into the car’s wing mirrors and rear-view mirror to peruse their environment. The rear view was provided by a combination of a back projection screen behind the cab (rear view mirror) and small monitor screens (door mirrors). The driving simulator vehicle parameters are; 1.67 m (5.5 ft) wide (center line of car ±, 0.83 m; 2.75 ft), 3.66 m (12 ft) long with a maximum speed of 177 km/h (110 mph), information was captured at 60 frames/second. Engine sound effects (braking, cornering tyre screech, and horn) were all turned on. The crash alarm was turned off, with the drive continuing as normal without reposition or reset of speed if participants collided with objects in their pathway. The triggering of events was carefully programmed to occur at the same distance into the drive for all participants, regardless of their speed.

#### The Drive

**FIGURE 2:**
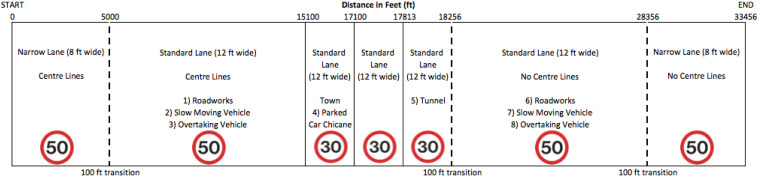
Visual representation of the main drive (not to scale) based on distance (in feet) including width of lane, road markings, event number (in order of which it occurred) and speed limit.

**FIGURE 3:**
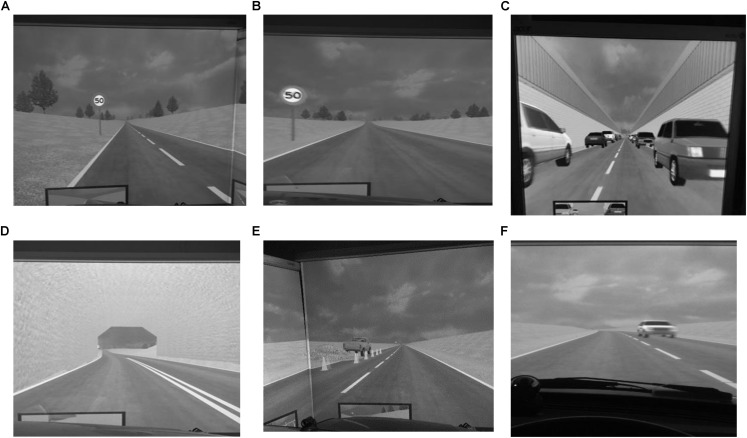
Photos of sections of drive for experiment 1 **(A–D)**, experiment 2 **(E)**, and experiment 3 **(F)**. **(A)** Photo of section of drive with central road markings. **(B)** Photo of section of drive without central road markings. **(C)** Photo of section of drive through narrow aperture. **(D)** Photo of section of drive through tunnel. **(E)** Photo of section of drive past roadworks. **(F)** Photo of section of drive past on-coming vehicle.

The drive (Figure 2) was 10.20 km (33,456 ft) in distance and took approximately 8–10 min to complete. The drive began in an 80.47 km/h (50 mph) zone with edge and center line markings on a single carriageway (2.42 m, 8-ft lanes) with steep grass banks. There are several shallow bends before the road widens (3.66 m, 12-ft lanes) and returns to being straight. Next, roadworks to the left of the road appear and some on-coming traffic. The road enters a town (48.3 km/h; 30 mph speed limit), passing between buildings with cars parked on either side of the road, opposite each other but separated by 3.66 m (12-ft). Next, the road exits the town between steep hills trees and enters a tunnel, curving to the left. On exit, the participants experience the same set of events they experienced previously but with no center line markings (80.47 km/h, 50 mph speed limit), starting with the 3.66 m (12 ft) wide lane and then 2.42 m (8 ft) wide lane. Photos of these different sections of the drive are presented in Figure 3.

### Procedure

Each participant completed the Adult DCD checklist and the Conners Adult ADHD Scales at home. Once seated in the driving simulator, participants were informed (a) that the car was automatic (accelerator and brake pedals indicated), (b) there was no need to use the handbrake or indicators, (c) they should adjust the rear-view mirror (with help if necessary), (d) the location of the speedometer (checked for recognition), and (e) they should treat all drives as a real driving situation and obey driving laws. Following answering any participant’s questions, the orientation drive was performed (3.3 m/10,800 ft in distance lasting 3–4 min). Participants then completed the main drive (10.2 m/33,456 ft in distance, lasting approximately 10 min). Upon completion of the main drive, participants completed the MABC-2, and WAIS-IV. Breaks were given as required throughout the testing procedure, which lasted approximately 2 h in total.

### Variables Measured

#### Longitudinal Speed

Longitudinal speed measured in miles per hour (mph) and converted to kilometers per hour (km/h).

#### Steering Adjustments

Steering adjustments were calculated by transforming the “Steering raw counts” to standardize the starting point for all participants to be zero. Steering raw counts is the raw output of the Analog to Digital converter on the steering sensors, this is presented in arbitrary units. The zero point is set when the simulation software is launched, but “straight on” is set as whatever position the steering wheel is in when the simulation run is launched. As, in almost all cases, the participant will move the steering wheel when seating themselves in the car, and although they will be asked to center the wheel before the simulation run is started, they will almost certainly not return the wheel to exactly the same place, hence the offset. The differences between each captured frame were calculated (as a series of points at 0.03 s intervals, which correspond to the video frames generated by the simulator), with any negative numbers transformed to the absolute value of the number before calculating the mean (the greater the rate of change of steering, the greater movement of the steering wheel).

#### Road Positioning

Road positioning measured in feet (ft), converted to meters (m) and taken from the mid-point of the car, in relation to the center line of the road (center line value = 0). Participants start the drive at −1.8 m (−6 ft) left of center line in left-hand lane). The left-hand curb is −3.65 m (−12 ft) and a positive result indicates the participant has crossed the center line onto right-hand side of road.

#### Collisions Number

Collisions number of times the driving simulator virtually “hit” an object in the pathway (taken for Conditions 1 and 2 only).

#### Latitudinal Movement

Latitudinal movement mean speed of latitudinal movement, measured in feet per second (ft/s) and converted to meters per second (m/s) (taken for Conditions 2 and 3 only).

## Condition Description and Measurement Parameters

### Condition 1 (Clear, Straight Road, and No Oncoming Traffic)

Condition 1 investigated behaviors of drivers and non-drivers with and without DCD as they negotiated a series of 4 driving scenarios of low but increasing perceptual load (1: straight road/central lines, 2: Straight road/no central lines, 3: Parked car, and 4: Tunnel). Please see Figures 3A–D for visual representation of this condition. Mean data were collected for each Scenario as follows; Scenario 1; 0–4.6 km (0–15,100 ft); Scenario 3 = 4.6–5.2 km (15,100–17,100 ft) Scenario 4 = 5.4–5.6 km (17,813–18,256 ft); Scenario 2 = 5.6–10.2 km (18,256–33,456 ft).

#### Question

Do drivers and non-drivers with and without DCD differ in their behaviors when negotiating driving scenarios with a low, but increasing, perceptual load?

### Condition 2 (Clear, Straight Road, No Oncoming Traffic, Stationary Obstacle in Pathway – Roadworks Straddling the Left Curb)

Condition 2 built upon the results of Condition 1 by increasing the motoric demands of the driving task whilst maintaining a low perceptual load. The task chosen reflected a scenario frequently encountered during the driving task and participants drove around roadworks positioned on the curb side of the road. The roadworks were positioned 1.98 km (6,500 ft) from the beginning of the drive and ended 2 m (6,570 ft) into the drive, extending 1.8 m (6 ft) from the curbside into the main carriageway. Pictorial representation of this scenario can be seen in Figure 3E.

#### Question

Do drivers and non-drivers with and without DCD differ in their behaviors when negotiating obstacles at the side of the road?

### Condition 3 (Clear, Straight Road, With Dynamic Object – Oncoming Traffic in Right Hand Lane)

Condition 3, further, increased the perceptual load as participants negotiated safe passage alongside an approaching vehicle on the opposite side of the road. The oncoming car was 3.05 m (10 ft) long and 1.83 m (6 ft) wide and traveled at a fixed speed of 48.3 km/h (30 mph) along the center of the righthand carriageway. It was created (and started traveling along the carriageway) when the driver reached 1.52 km (5,000 ft) into the drive, 0.5 km (1,500 ft) ahead of the driver. Please see Figure 3F for visual representation of this condition.

#### Question

Do drivers and non-drivers with and without DCD differ in their behaviors as they pass an oncoming vehicle on the opposite side of the road?

### Data Extraction for Condition 2 and 3

For Condition 2, the data were extracted ±1 s from the point where the driver encountered the roadworks on a participant-by-participant basis in order to account for differing velocities. This was accomplished by specifying the point on the drive when the object was created, the distance from the driver, and the velocity at which the object was moving toward the driver (in this case 0 m/s; O ft/s). Using these variables, it was possible to calculate the distance of the object from the driver as they navigated the simulation and identify when they passed each other. The data for Condition 3 were extracted in the same way as in Condition 2, but in this case the obstacle was not static and so the velocity at which it approached the driver was set at a constant 9.1 m/s (30 ft/s).

## Data Analysis

For all analyses, significant interactions were explored using simple main effects, and significant main effects were investigated using planned comparisons. Bonferroni corrections were applied to protect against Type I error. Mauchly’s test of Sphericity was violated and Greenhouse-Geisser correction was therefore applied to all interactions. Please see Table 2 for mean values by condition, driving experience and group.

**TABLE 2:** Mean values (*SD*) for speed (km/h/mph), road position (m/ft) steering adjustment, lateral speed (mps/fps) and collisions by condition (1, 2, and 3), and driving status (driver: non-driver) and group (DCD:TD).

		DCD		TD	

Speed km/h (mph)	Condition	DCD:D	DCD:ND	TD:D	TD:ND
1. No central lines	1	67.9 (42.2) (7.4)	65.5 (40.7) (2.6)	72.1 (44.8) (3)	68.8 (42.7) (3.4)
2. Central lines		68.7 (42.7) (6.3)	65.7 (40.8) (3.1)	73.1 (45.4) (3.6)	66.5 (41.3) (4.5)
3. Parked cars		31.1 (19.3) (6)	43.0 (26.7) (9.3)	40.2 (25) (5.3)	40.1 (25) (8.2)
4. Tunnel		44.8 (27.8) (2.5)	39.3 (24.4) (2.7)	43.5 (27.0) (2.3)	40.5 (25.2) (2.8)
5. Roadworks	2	61.9 (38.5) (4.6)	61.4 (38.2) (3.0)	64.7 (40.2) (3.6)	61.9 (38.5) (3.6)
6. Oncoming vehicle	3	69.3 (43.1) (4.9)	64.2 (39.9) (3.2)	73.5 (45.7) (3.8)	65.8 (40.9) (4.85)

**Road Position m (ft)**		**DCD:D**	**DCD:ND**	**TD:D**	**TD:ND**

1. No central lines	1	**−**1.28 (**−**4.2) (1.1)	**−**1.06 (**−**3.5) (1.7)	**−**1.31 (**−**4.3) (0.6)	**−**1.28 (**−**4.2) (1.2)
2. Central lines		**−**1.49 (**−**4.9) (0.5)	**−**1.46 (**−**4.8) (1)	**−**1.58 (**−**5.2) (0.4)	**−**1.58 (**−**5.2) (0.8)
3. Parked cars		**−**0.57 (**−**1.9) (0.8)	**−**0.67 (**−**2.2) (0.9)	**−**0.49 (**−**1.6) (0.7)	**−**0.67 (**−**2.2) (0.8)
4. Tunnel		**−**1.85 (**−**6.1) (0.8)	**−**1.8 (**−**5.9) (1)	**−**1.89 (**−**6.2) (0.6)	**−**1.95 (**−**6.4) (1.1)
5. Road works	2	**−**1.85 (**−**6.1) (0.88)	**−**1.89 (**−**6.2) (1.47)	**−**1.92 (**−**6.3) (0.66)	**−**2.1 (**−**6.8) (0.81)
6. Oncoming vehicle	3	**−**2.28 (**−**7.5) (1.18)	**−**2.24 (**−**7.4) (1.78)	**−**2.34 (**−**7.7) (0.57)	**−**2.43 (**−**8) (1.04)

**Steering Adjust**		**DCD:D**	**DCD:ND**	**TD:D**	**TD:ND**

1. No central lines	1	3.8 (1.3)	4.6 (3.2)	3.8 (1.2)	3.7 (1.8)
2. Central lines		4.2 (0.6)	4.5 (1.8)	4.2 (0.9)	4.3 (2.1)
3. Parked cars		8.4 (11.8)	9.2 (4.4)	6.6 (2.9)	6.7 (7.3)
4. Tunnel		26 (8.1)	17.4 (5.9)	20.5 (8.7)	20.5 (10.4)
5. Road works	2	4.7 (2.4)	4.9 (2.1)	4.2 (1.7)	4.03 (1.4)
6. Oncoming vehicle	3	31 (26.7)	42.7 (50.8)	22.8 (18.4)	26.6 (34.8)

**Lat Speed m/s (fps)**		**DCD:D**	**DCD:ND**	**TD:D**	**TD:ND**

5. Road works	2	**−**0.4 (**−**1.2) (1.0)	**−**0.5 (**−**1.6) (1.2)	**−**0.4 (**−**1.3) (0.9)	**−**0.3 (**−**0.9) (1.2)
6. Oncoming vehicle	3	0.03 (0.11) (0.4)	**−**0.04 (**−**0.13) (0.5)	**−**0.04 (**−**0.13) (0.3)	0.3 (0.9) (0.4)

**Collisions**		**DCD:D**	**DCD:ND**	**TD:D**	**TD:ND**

	1	0.2 (0.4)	3.9 (7)	0.1 (0.3)	2.1 (3.4)
	2	0 (0)	0 (0)	0 (0)	0.6 (1.2)

### Condition 1

Four separate repeated measures Analysis of variance (ANOVA) were used to investigate the effect of Group (DCD; TD), Driver Status (driver; non-driver) and Scenario (1: Lines; 2: No lines; 3: Narrow aperture/parked cars; and 4: Tunnel) on speed, road positioning, steering adjustments, and collisions.

### Conditions 2 and 3

Separate ANOVAs were conducted to investigate the effect of Group (DCD; TD), and Driver Status (driver; non-driver) on speed, road positioning and steering adjustments, collisions as well as latitudinal movement for the period of time when the car was driving past the roadworks (Condition 2: static obstacle) and vehicle driving toward them (Condition 3 dynamic obstacle, velocity at which it approached the driver was set at a constant 33 km/h (30 ft/s).

## Results

### Condition 1 Behaviors of Drivers and Non-drivers With and Without DCD as They Negotiate a Clear, Straight Road in 4 Scenarios of Increasing Perceptual Complexity

#### Speed (km/h/mph)

There was a significant effect for Scenario [*F*(3,81) 296.86, *p* ≤ 0.001, η*p*^2^ = 0.853]. Parameter estimates and contrast analysis reveal that participants drove slower in both the parked car (39 km/h/24 mph) and the tunnel (42 km/h/26 mph) scenarios compared to the lines (69 km/h/42 mph) and no-lines scenarios (69 km/h/43 mph). A Scenario-by-Driver Status interaction [*F*(3,81) 6.956, *p* = 0.003, η*p*^2^ = 0.120] revealed that drivers drove faster than non-drivers in all scenarios except parked cars, where they were slower. However, observations of the mean values in Table 2 highlight that this significant interaction is mainly due to the drivers with DCD and a lack of a group interaction here might be due to low power. To investigate this further, planned comparisons using Mann-Whitney-U were run and showed that drivers with DCD were significantly slower in the parked car Scenario (*M* = 31 km/h/19.32 mph) compared to TD drivers (*M* = 40.2 km/h/24.99 mph), *U* = 129, *p* = 0.027.

#### Road Position (ft/m)

A significant effect for Scenario [*F*(3,129), 292.027, *p* < 0.001, η*p*^2^ = 0.851], together with mean values and pairwise comparisons revealed that road positioning was significantly different between each scenario (*p* < 0.001, M No-lines = **−**1.25 m/**−**4.11 ft; M Lines = **−**1.54 m/**−**5.04 ft; M Parked car = **−**0.6 m/**−**1.97 ft; M Tunnel = **−**1.9 m/**−**6.23 ft).

#### Steering Adjustment

A significant effect for Scenario [*F*(3,96), 119.317, *p* < 0.001, η*p*^2^ = 0.701], together with mean values and pairwise comparisons revealed that all participants used more steering adjustments in the parked car scenario (M Parked car = 7.37) compared to the other scenarios (M No-lines = 3.91, *p* = 0.004; M Lines = 4.29, *p* = 0.001; M Tunnel = 4.37, *p* = 0.010).

#### Collisions

There was a significant effect of Driver Status [*F*(3,51) 8.490, *p* = 0.005, η*p*^2^ = 0.142] showing that non-drivers had significantly more collisions (*M* = 2.68; *SD* = 4.78) than drivers (*M* = 0.14; *SD* = 0.36) throughout the drive. Further investigation of mean values reveals that this effect is mainly due to non-drivers with DCD who had more collisions (*M* = 3.89; *SD* = 6.99) than the TD non-drivers (*M* = 2.10; *SD* = 3.38).

#### Summary of Findings for Condition 1

Condition 1 investigated the effects of Group, Driver Status and Scenario on speed, road positioning, steering adjustments and collisions whilst driving under a low, but increasing perceptual load along a level, straight roadway. Analyses revealed that all drivers responded to increases in perceptual load, reducing their speed as they negotiated the parked car and tunnel scenario and used more steering adjustments whilst driving through the narrow aperture created by the parked car scenario. However, it appears that drivers with DCD may have been more affected by the increase in perceptual load as the Scenario-by-Driver Status interaction shows that drivers with DCD drove more slowly than TD drivers in the parked car scenario. Analysis also revealed that non-drivers had significantly more collisions than drivers. Finally, all participants adjusted their road position to accommodate the task demands. For example, whilst it is unsurprising that participants drove more centrally in the parked car scenario (**−**0.6 m/**−**1.97 ft), they also positioned the car more centrally in the left-hand lane in the tunnel scenario (**−**1.9 m/**−**6.23 ft) compared to the other scenarios (No-lines = **−**1.25 m/**−**4.11 ft; M Lines = **−**1.54 m–5.04 ft).

### Condition 2: Behaviors of Drivers and Non-drivers With and Without DCD as They Negotiate Static Obstacle (Roadworks) in the Pathway

#### All Measures

Condition 2 investigated the effects of Group and Driver Status on speed, road positioning, steering adjustments, collisions and latitudinal movement whilst negotiating roadworks on the curb side of a level, straight roadway. There were no significant main effects or interactions for Speed (all *F*s < 1.6, all *p*s > 0.2), Road position (all *F*s < 2.6, all *p*s > 0.1), Steering adjustment (all *F*s < 2.4, all *p*s > 0.1), Collisions (all *F*s < 2.2, all *p*s > 0.1), and latitudinal movement (all *F*s < 1.3, all *p*s > 0.2). Drivers and non-drivers with and without DCD drove at a similar speed and road position, they used a similar number of steering adjustments, collisions and latitudinal movement as they negotiated the roadworks. These findings show that increasing the motoric demands of a task with a low perceptual load for drivers and non-drivers with and without DCD had little effect on behavior.

### Condition 3: Behaviors of Drivers and Non-drivers With and Without DCD as They Negotiate Oncoming Traffic

#### Speed (km/h/mph)

There was a significant effect for Driver Status [*F*(3,51) 8.334, *p* = 0.006, η*p*^2^ = 0.140] showing that drivers drove faster (71.3 km/h/44.30 mph) than non-drivers (65.1 km/h/40.45 mph) when passing a moving vehicle on the opposite side of the road.

#### Road Position (m/ft) and Steering Adjustments

There were no significant effects for Road position (all *F*s < 1.9, all *p*s > 0.1) or Steering adjustment (all *F*s < 2, all *p*s > 0.1),

#### Latitudinal Movement (mps/fps)

**FIGURE 4:**
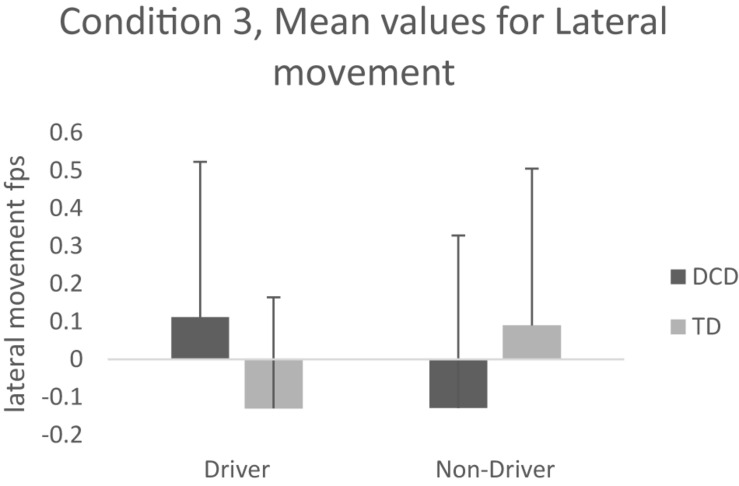
Mean values for lateral movement condition 3.

A significant Group-by-Driver Status interaction [*F*(3,51), 4.388, *p* = 0.041, η*p*^2^ = 0.079], mean values and pairwise comparisons show that lateral movement was significantly different between drivers with and without DCD (*p* = 0.045). Drivers with DCD drove further to the right, veering toward the oncoming vehicle (+0.03 m/s/ +0.112 fps) whereas TD drivers drove further to the left, veering away from the oncoming vehicle (**−**0.04 m/s/- 0.130 fps). This effect was not evident when comparing non-drivers in the DCD and TD groups. Please see Figure 4. for illustration.

#### Summary Condition 3

Condition 3, investigated the effects of Group and Driver Status on speed, road positioning, latitudinal movement, and steering adjustments while passing an approaching vehicle on the opposite side of the road. Drivers drove faster than non-drivers when passing a moving vehicle on the opposite side of the road and the Group-by-Driver Status effect for latitudinal movement, showed that drivers with DCD veered toward (to the right) the oncoming car, whereas the TD drivers veered away from it (to the left).

## Discussion

The main aim of this study was to investigate behaviors of drivers and non-drivers with and without DCD when negotiating everyday scenarios. We used a series of three conditions to gradually increase the perceptual load and answer the following research questions; (1) Do drivers and non-drivers with and without DCD behave differently when processing dynamic sensory information in progressively complex environments? (2) How do drivers and non-drivers with and without DCD negotiate narrow apertures in a car of fixed width? (3) Do individuals with DCD collide more with obstacles in the pathway compared to controls? Given previous research (Cousins and Smyth, 2003; de Oliveira and Wann, 2011, 2012; Wilmut et al., 2015; Gentle et al., 2016), we expected that, across all conditions compared to controls, individuals with DCD would drive more slowly, have less appropriate road positioning, use more steering adjustments, and have more collisions. We also predicted that group differences would be more pronounced in conditions with higher perceptual load.

Whilst there were no main effects for group for any of the conditions, and our main hypothesis cannot be fully supported, several significant interactions show that individuals with and without DCD respond differentially to changing perceptual loads when negotiating everyday driving scenarios. For example, in condition 1, drivers with DCD were significantly slower in the parked car scenario compared to all other groups (non-drivers with DCD, TD drivers, and TD non-drivers). Furthermore, in Condition 3 drivers with DCD (but not non-drivers) drove further to the right, veering toward the oncoming vehicle whereas TD drivers drove further to the left, veering away from the oncoming vehicle. These behaviors suggest that TD drivers allow a larger gap between themselves and the oncoming vehicle to accommodate the additional demands of the task and maximize the opportunity for safe passage alongside the vehicle. The drivers with DCD do not adopt this ‘safer’ strategy and actually put themselves in greater danger by veering toward the oncoming vehicle.

It is beyond the remit of this study to identify causality, but differences in behaviors whilst negotiating oncoming traffic could be related to quality of visuo-motor integration. There is much evidence in the literature suggesting that vision and the motor control needed for steering are strongly interconnected (Land and Lee, 1994). Visual information of the road informs the arm-motor system controlling the steering wheel (Mars and Navarro, 2012), providing a direct link between gaze direction and steering (Robertshaw and Wilkie, 2008). Wilkie et al. (2008) showed that we steer where we look, and look where we steer (Land, 1998). These findings are supported by professional-but-anecdotal advice given in the Police Drivers Handbook and Experienced Rider Course (for motorcyclists) which warns against looking directly at a hazard to prevent steering toward it. Thus, if drivers with DCD are failing to inhibit the link between eye gaze direction and steering, this could explain why they veer toward the oncoming vehicle, placing them in a less optimal road position compared to the other groups in this study.

There may also be a biomechanical explanation related to postural control which is a fundamental skill necessary for every movement we make. Like all movement, postural control is heavily dependent upon the efficient integration of visual, vestibular, and proprioceptive information (Oie et al., 2002), an area of known difficulty for individuals with DCD (Wilson et al., 2013). There are two different strategies to control posture; “enbloc” (movement of head and trunk together) and ‘articulated’ (head is stabilized at the neck separately from the trunk, Assaiante and Amblard, 1995) argue that. The articulated mode of head stabilization occurs developmentally as sensitivity to the orientation of the head about the trunk increases, together with increased ability to control the degrees of freedom about the neck and head (Lund and Broberg, 1983). Assaiante and Amblard (1995) argue that this model predicts that any impairment in sensorimotor or biomechanical systems may prevent or postpone development. If the drivers with DCD are adopting the “en bloc” strategy to stabilize visual and vestibular information, this might explain why they veer toward the oncoming car. As the individuals with DCD move their eyes toward the oncoming vehicle, the head and trunk (and arms) follow “en bloc,” steering the car toward the oncoming vehicle.

It is of note that in several scenarios, the non-drivers with DCD behave more like the TD groups than the drivers with DCD. For example, in Condition 1 the non-drivers with DCD drove at a similar speed to the TD drivers in the more challenging scenario when negotiating the parked cars. This behavior is replicated in Condition 3 where non-drivers with DCD and the TD controls veer away from the oncoming vehicle. There is no evidence to suggest that the non-drivers with DCD can perceive the dynamic information more accurately than the drivers with DCD, so what is happening in these scenarios to explain these inter-group differences?

An explanation can be sought after reviewing the data in Table 2. Drivers with DCD have higher standard deviations, particularly for speed and steering adjustments, suggesting more variance in their driving ability. The standard deviations for the non-drivers with DCD are lower and so they are more consistent. Given the age profiles of the participants for this study, it is possible that non-drivers with DCD and the TD groups have similar levels of driving experience as they have similar mean ages (21 years) compared to the drivers with DCD who had a higher mean age and wide age range (32 year, *SD* = 11.7). Thus, it could be that the wide age range reflects differences in driving experience which implicates performance. Clearly, there is a need for more investigation to tease apart the mechanisms behind differing behaviors for drivers and non-drivers with DCD.

A lack of significant effects for any of the measures taken for Condition 2 suggests that a small increase in motoric (but not perceptual) load of the task does not implicate behaviors for drivers or non-drivers with and without DCD. Whilst non-significant group effects are positive in terms of perceptions of the driving task, they need further discussion given previous work in this area. Firstly, individuals with DCD drove alongside the roadworks at a similar speed to their TD peers, supporting the findings in both the de Oliveira and Wann (2011, 2012) driving studies. Furthermore, all participants slowed down (compared to the low perceptual load scenarios 1& 2 in Condition 1) as they negotiated the roadworks, suggesting appropriate awareness of the obstacle in the pathway.

However, the lack of group difference in road position was not expected, as previous work investigating navigation of obstacles in the pathway (Wilmut et al., 2015) suggests that individuals with DCD allow a higher “margin of error” to accommodate issues with visuo-motor integration and avoid collision. Methodological differences between studies may account for these unexpected results. For example, the Wilmut et al. (2015) study involved a narrow aperture which was constrained on both sides rather than only one side as in the current experiment. Had there been any oncoming traffic to limit the aperture width, behaviors of the DCD group may have been different. We also expected that individuals with DCD would use more steering adjustments, compared to the TD group when negotiating the roadworks (de Oliveira and Wann, 2011, 2012). Differences here can be explained in terms of task complexity as it could be argued that the motoric demands to safely negotiate a bend, as in the de Oliveira studies, are higher than avoiding roadworks protruding only 1.8 m (6 ft) into a 3.6 m (12 ft) carriageway found in the current study.

It must also be noted that, the measures taken in Condition 2 may not have been sensitive enough to reflect the subtlety of the sensory and motoric processes needed to successfully negotiate the roadworks (Cloete and Wallis, 2009). Future work could focus on a more detailed analysis of the specific demands of the task as a driver initiates steering to avoid the object in the pathway, then center’s the vehicle on the straight trajectory, and finally repositions the vehicle on the main carriageway after completing the maneuver (Hildreth et al., 2000). Findings from this work would inform the literature on the contribution of group and driver status to navigational skills in complex, but real-world environments.

In terms of Driver status, we expected that non-drivers would drive more slowly and have more collisions than drivers. We can accept this hypothesis as results from Condition 3 show that non-drivers drove more slowly than drivers as they negotiated the oncoming vehicle and in Condition 1, non-drivers had more collisions than drivers. These findings reflect many studies in the literature of a greater incidence of crash rate for low mileage/inexperienced drivers compared to higher mileage drivers (e.g., Langford et al., 2006; Antin et al., 2017). Indeed, McCartt et al. (2009) show that when the relative importance of age and experience are investigated, it is experience that has the greatest effect on crash frequency. However, as this study did not measure driving experience, we need to interpret these findings with caution. Indeed, future work investigating the effect of experience on the success of the driving task would benefit the literature on driving with DCD. We know that individuals with DCD take longer to pass their test (Missiuna et al., 2008) and therefore gain more driving experience as they learn to drive compared to their TD peers. However, we do not know how this experience translates to driving skill.

### Implications of This Research

We know that inexperienced drivers are more susceptible to road traffic accidents (Langford et al., 2006; McCartt et al., 2009; Antin et al., 2017) and for individuals with DCD, the additional perceptual constraints add a substantial cognitive load to the driving task (Wann et al., 1998; Missiuna et al., 2008; de Oliveira and Wann, 2011, 2012; Wilson et al., 2013). Thus, an individual’s sensitivity to information from the environment needs to be considered alongside personal constraints that may affect how that information is used to inform movement and the driving task. One of the main implications for this work is to recognize the impact of both internal and external constraints on the ability of individuals with and without DCD to successfully interact with the environment. There is still much work to be explored in this area to fully understand the mechanisms behind skillful driving.

Interventions to support individuals with DCD when learning to drive would benefit from an environment with a low perceptual load such as off-road provision or an empty carpark to allow the individual with DCD to learn the motoric demands of the driving task. This strategy will provide a safe, yet effective approach and the individual can then slowly be introduced to a more dynamic environment as ability and confidence increase. Furthermore, the use of an automatic car would reduce the motoric demands of gear changing whilst driving to allow more attention to be given to the perceptual demands of the task.

## Conclusion

We argue that drivers and non-drivers with and without DCD do behave differently when driving in progressively complex environments. However, the data presented here suggests differences within the DCD group need further investigation to fully understand the mechanisms that contribute to driving behaviors for this population. This study highlights how drivers and non-drivers with and without DCD apply a variety of strategies to accommodate their personal constraints depending on their perception of the task and environmental conditions. It is therefore important to consider both group membership, and driver status when evaluating behaviors whilst driving. The evidence suggests that even within a very safe and predictable environment (level, uncluttered road, and no unexpected hazards), as the perceptual demands of the tasks increased, individual constraints (lack of experience/difficulties in perceiving and responding to the environment) influenced the efficiency with which participants can respond to the dynamic environment when driving. These findings reflect previous suggestions of a deficient mapping between visual information and steering actions for individuals with DCD (Fajen, 2008; de Oliveira and Wann, 2012) and provide quantitative evidence to support work by Missiuna et al. (2008) and Kirby et al. (2011) who found that individuals with DCD self-reported particular difficulty with more complex driving skills, such as parking or reversing.

## Data Availability Statement

The datasets presented in this study can be found in online repositories. The names of the repository/repositories and accession number(s) can be found below: Open Science Framework (https://osf.io/frv29/).

## Ethics Statement

The studies involving human participants were reviewed and approved by the University of Surrey FHMS Research Ethics Committee. The patients/participants provided their written informed consent to participate in this study.

## Author Contributions

JG, SC, and NW designed the study and undertaken data collection. NW and DB extracted driving data. JG and DB completed statistical analysis. JG completed Table and Figure preparation and first draft of the manuscript. JG, HL, and DB edited the manuscript. All authors approved the final version of this manuscript.

## Conflict of Interest

The authors declare that the research was conducted in the absence of any commercial or financial relationships that could be construed as a potential conflict of interest.
